# CAN HEART DISEASE SELF-MANAGEMENT EDUCATION ALSO REDUCE PAIN INTERFERENCE IN AFRICAN AMERICAN OLDER ADULTS?

**DOI:** 10.1093/geroni/igz038.2634

**Published:** 2019-11-08

**Authors:** Mary R Janevic, Kristi Allgood, Jessica Ramsay, Cainnear Hogan, Rebecca Courser, Cathleen M Connell

**Affiliations:** University of Michigan School of Public Health, Ann Arbor, Michigan, United States

## Abstract

Many older adults with heart disease, especially those from vulnerable populations, live with chronic pain. Self-management strategies for reducing cardiovascular risk (e.g., physical activity, stress reduction) are also recommended for management of chronic pain. It is not known, however, whether self-management education focused on heart health has a beneficial “side effect” on pain-related outcomes. We explored this possibility using data from a randomized controlled trial (n=405) of Take Heart, a group intervention adapted for low-income, African American adults 50 and over with heart disease or significant risk factors. We first assessed the sample prevalence of high-impact chronic pain; i.e., substantial pain-related disability in one or more life domains (National Pain Strategy, 2016). Next, we assessed whether participation in Take Heart resulted in decreased pain interference (PROMIS-29). One-third of participants (n=131) met criteria for high impact chronic pain. Mean pain interference T-score in the entire sample at baseline was 58.1 (SD=9.9), indicating a score one standard deviation greater than population average. Compared to controls, intervention participants had a greater, but non-significant, improvement in pain interference (0.7 vs. -0.8; p=.11). Overall, findings demonstrate that African American adults with cardiovascular conditions have a high burden of comorbid pain. This pain was not greatly improved by an intervention that taught key chronic disease self-management skills but did not address pain specifically. Future research can test whether incorporating pain-management content into heart disease education has a stronger impact on improving pain outcomes -- which may, in turn, promote adherence to behaviors to reduce cardiovascular risk.

